# iMir: An integrated pipeline for high-throughput analysis of small non-coding RNA data obtained by smallRNA-Seq

**DOI:** 10.1186/1471-2105-14-362

**Published:** 2013-12-13

**Authors:** Giorgio Giurato, Maria Rosaria De Filippo, Antonio Rinaldi, Adnan Hashim, Giovanni Nassa, Maria Ravo, Francesca Rizzo, Roberta Tarallo, Alessandro Weisz

**Affiliations:** 1 Laboratory of Molecular Medicine and Genomics, Department of Medicine and Surgery, University of Salerno, via Allende, 1, Salerno, Baronissi, Italy; 2 Fondazione IRCCS SDN, Napoli, Italy; 3 Division of Molecular Pathology and Medical Genomics, “SS. Giovanni di Dio e Ruggi d’Aragona – Schola Medica Salernitana” University of Salerno Hospital, Salerno, Italy

**Keywords:** Next generation sequencing, SmallRNA-Seq, Data analysis pipeline, Breast cancer, Small non-coding RNA, microRNA, Piwi-interacting RNA

## Abstract

**Background:**

Qualitative and quantitative analysis of small non-coding RNAs by next generation sequencing (smallRNA-Seq) represents a novel technology increasingly used to investigate with high sensitivity and specificity RNA population comprising microRNAs and other regulatory small transcripts. Analysis of smallRNA-Seq data to gather biologically relevant information, i.e. detection and differential expression analysis of known and novel non-coding RNAs, target prediction, etc., requires implementation of multiple statistical and bioinformatics tools from different sources, each focusing on a specific step of the analysis pipeline. As a consequence, the analytical workflow is slowed down by the need for continuous interventions by the operator, a critical factor when large numbers of datasets need to be analyzed at once.

**Results:**

We designed a novel modular pipeline (iMir) for comprehensive analysis of smallRNA-Seq data, comprising specific tools for adapter trimming, quality filtering, differential expression analysis, biological target prediction and other useful options by integrating multiple open source modules and resources in an automated workflow. As statistics is crucial in deep-sequencing data analysis, we devised and integrated in iMir tools based on different statistical approaches to allow the operator to analyze data rigorously. The pipeline created here proved to be efficient and time-saving than currently available methods and, in addition, flexible enough to allow the user to select the preferred combination of analytical steps. We present here the results obtained by applying this pipeline to analyze simultaneously 6 smallRNA-Seq datasets from either exponentially growing or growth-arrested human breast cancer MCF-7 cells, that led to the rapid and accurate identification, quantitation and differential expression analysis of ~450 miRNAs, including several novel miRNAs and isomiRs, as well as identification of the putative mRNA targets of differentially expressed miRNAs. In addition, iMir allowed also the identification of ~70 piRNAs (piwi-interacting RNAs), some of which differentially expressed in proliferating *vs* growth arrested cells.

**Conclusion:**

The integrated data analysis pipeline described here is based on a reliable, flexible and fully automated workflow, useful to rapidly and efficiently analyze high-throughput smallRNA-Seq data, such as those produced by the most recent high-performance next generation sequencers. iMir is available at http://www.labmedmolge.unisa.it/inglese/research/imir.

## Background

Small RNA analysis by massively parallel sequencing (smallRNA-Seq) represents an increasingly popular method to address different questions concerning the biological role of miRNAs and other regulatory small transcripts, such as piwi-interacting (piRNAs), small inhibitory (siRNAs), transcription initiation (tiRNAs), transfer (tRNAs) and other small non-coding (sncRNAs) RNAs, including also extra-cellular small RNAs (exRNAs). Among sncRNAs, miRNAs and piRNAs are emerging as key regulators in multiple cellular functions and for this reason are widely studied by direct sequencing. miRNAs, the best know and studied class of sncRNAs, are interesting to investigate due to their ability to control gene expression in eukaryotes by fine tuning mRNA translation [1-3]. They represent a class of short (~ 22 nucleotides) RNA molecules that play pivotal roles in a variety of molecular processes, such as immune response [4], differentiation [5], development [6-8], infection [9,10] and carcinogenesis [11-13]. miRNA genes are synthesized as long precursor RNA molecules (pri-miRNAs), usually by RNA polymerase II [14], that are rapidly processed in the nucleus by Drosha RNase III to release approximately 70 nucleotides long miRNA precursor stem loop (pre-miRNA) [15] that in turn are exported to the cytoplasm by Exportin 5 [16]. In the cytoplasm, mature miRNAs are produced through the action of Dicer RNase [17]. These small RNAs regulate gene expression by binding to targets sites generally in the 3′ untraslated region (3′ UTR) of target mRNAs, resulting in mRNA degradation or translation inhibition [1,18]. miRNAs recognition of the 3′ UTR of their target mRNA is mediated by hybridization between nucleotides 2–8 at 5′ end of the small RNA (seed sequence) and the complementary sequences present in the 3′ UTR of the mRNA [1,19,20]. On the other hand, small non-coding RNAs that interact with Piwi proteins, called piRNAs, are emerging as regulatory transcripts able to control a broad range of biological processes. The main roles of these molecules has been investigated mainly in germline stem cells, where they are involved in: (i) regulation of transposone activity; (ii) modulation of genome epigenetic state, (iii) development and (iv) spermatogenesis [21]. However piRNAs have been also identified in somatic cells, including human cancer cells [22], suggesting their possible involvement in tumors. This aspect highlights the need for sensitive and efficient bioinformatics tools to study these novel class of sncRNAs in smallRNA-Seq datasets. SmallRNA-Seq allows detection of RNAs with a high dynamic range and reliably measures small differences in RNA concentration between samples, enabling also to discover novel RNA molecules not annotated in databases. Generally, data analysis is performed by combining multiple statistical and bioinformatics tools available from different sources. Many useful programs for processing these data exist nowadays, such as RandA [23], Shortran [24], UEA sRNA Workbench [25], DSAP [26], miRTools 2.0 [27] and miRExpress [28]. Two main issues hamper diffusion and implementation of such programs: (i) web-based tools have some restriction on data upload; (ii) stand-alone programs often lack one or more analysis steps, such as for example prediction of novel sncRNAs. As main consequence, the analytical workflow is slowed down by the need for the continuous interventions by the operator, a critical factor when a large number of samples need to be analyzed at once. A main challenge in bioinformatics is thus to create comprehensive computational tools for handling and analyzing, in an automated manner, the huge amount of data generated by these experiments.

We describe here a modular analysis pipeline, iMir, for comprehensive analyses of smallRNA-Seq data integrating multiple open source modules and resources linked together in automated way. The pipeline allows identification of miRNAs and other sncRNAs, such as piRNAs, to perform differential expression analysis and, for miRNAs, to predict the corresponding mRNA targets. In addition, iMir provides the possibility to perform hierarchical clustering and to apply different statistical approaches to the analysis, improving discrimination of expressed sncRNAs and allows to identify those more likely to be biologically relevant. The pipeline output includes graphics and text files that are useful for a better interpretation of the results. iMir is well suited for the analysis of smallRNA-seq data obtained from animal samples. Moreover, it can be used to investigate the role of sncRNAs in plants adding the appropriate reference tracks in iMir database.

## Implementation

**Figure 1:**
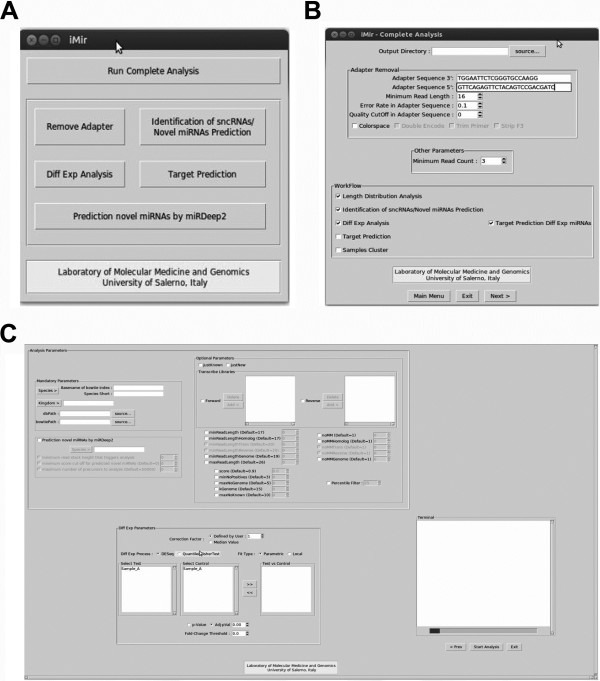
**iMir Graphical User Interface.** iMir GUI screen-shots. **A:** Once iMir is launched the user can define which step of the analysis perform and set different parameters for adapter cleavage. **B:** In the next step, the user can select and rename the samples, while the next windows **C:** allows to set parameter for detect known and novel miRNAs and differential expression analysis.

**Figure 2:**
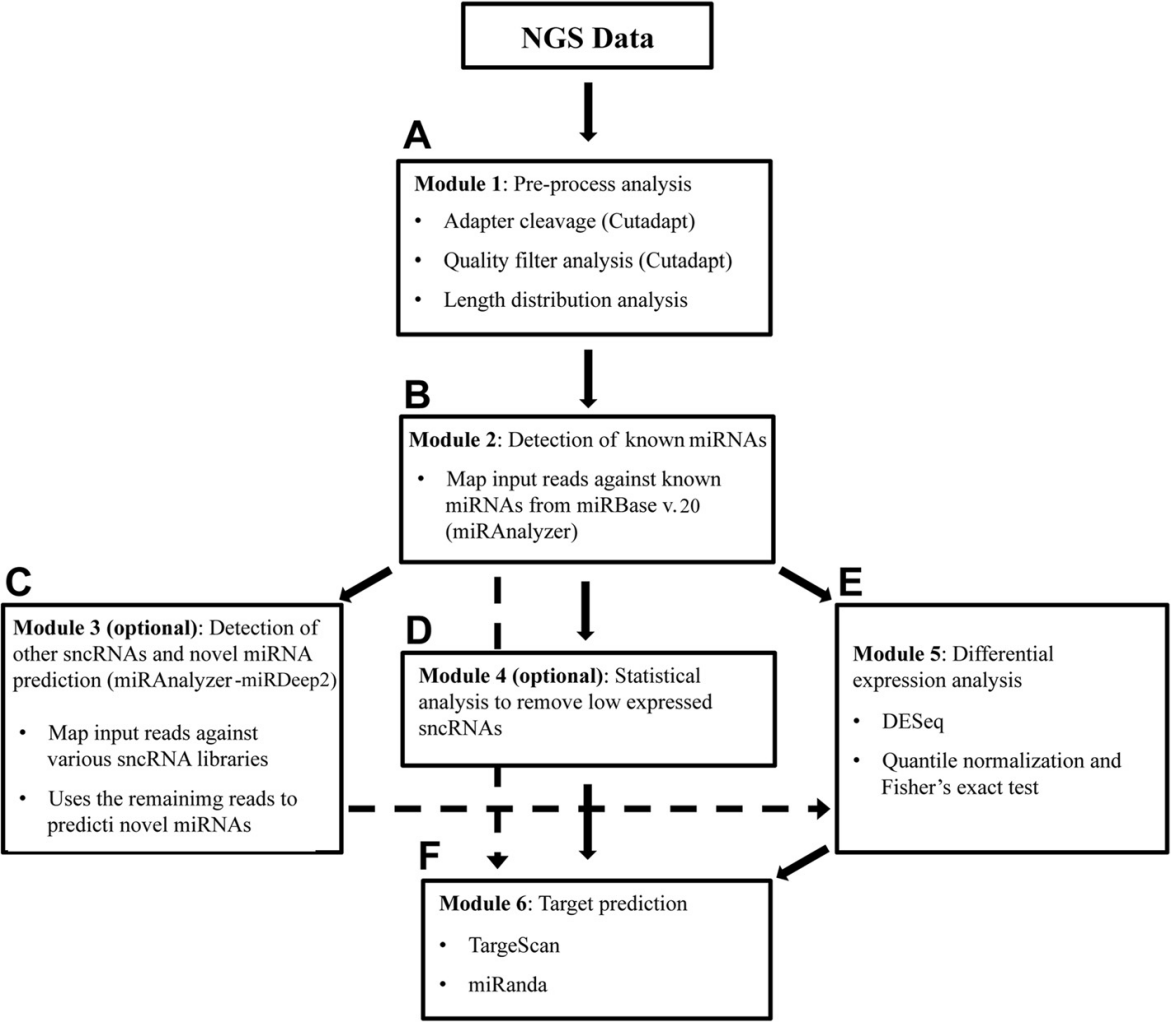
**iMir workflow.** Graphic summary of iMir workflow: the pipeline accepts NGS data as input and then proceeds automatically to perform several independent analyses, most of which can be selected or excluded according to the user’s needs. Dotted lines represent optional steps of the pipeline. **(A)** Module 1: Pre-process analyis. **(B)** Module 2: Detection of known miRNAs. **(C)** Module 3: Detection of other sncRNAs and novel miRNAs prediction. **(D)** Module 4: Statistical analysis to remove low expressed sncRNAs. **(E)** Module 5: Differential expression analysis. **(F)** Module 6: Target prediction.

One of the main problems when dealing with the large datasets generated with the currently available Next Generation Sequencing (NGS) technologies are represented by the difficulties in their management and analysis. Analysis of smallRNA-Seq data, for example, requires implementation of different bioinformatics tools and the possibility to perform multiple, subsequent file format conversions that slows-down and makes cumbersome the analytical procedure. The need of a bioinformatics instrument that may help solve these problems in a user-friendly and handy way led us to devise a tool, called iMir, that integrates various open source modules and resources and, in addition, implementing different statistical approaches for sncRNAs expression analyses allows users to select the most performing and relevant method for analysis of their data. The analytical modules included in iMir, selected among the best available, have been made more performant thanks to home-made scripts that allows the user to create self-defined analytical flows. Indeed, the resources integrated in iMir were selected after careful comparison and throughout evaluation of the performance of software widely used in smallRNA-Seq data analysis, according also to what recently reported by Cordero et al. [29] and Williamson et al. [30]. iMir was implemented using an object-oriented programming language, Python, and comprises also a Graphical User Interface (GUI, Figure 1A-C and Additional file 1) that makes it easier the use of command line tools, so that the pipeline is particularly suited for biologist and early stage bioinformaticians, also because it simplify the way how projects are created, parameters are specified for each of several steps of the analysis and the different algorithms are run on project data (Figure 1A). In addition, terminal display window (Figure 1C) can be used to follow the flow of analysis. iMir provides stepwise planning that allows user to select the desired combination of analytical tools in the workflow. Some modules are mandatory while others are, instead, optional. A schematic representation of the iMir workflow is shown in Figure 2A-F. The pipeline takes in input the deeply sequenced reads in FASTQ format. As mentioned above, iMir offers the possibility to run different modules independently and this can be considered one of its main advantages. Indeed, in some cases user can work using pre-analyzed datasets (e.g. reads clipped from adapters sequences, or table with read-counts for each sncRNAs detected in test *vs* control samples), or may need to perform only a specific analytical step, such as adapter cleavage from input reads, detection of known and/or novel miRNAs, or to map sequence reads against other sncRNA libraries and then to perform differential expression analysis. In all cases, it is possible to start the analytical flow at that step simply by using the input file specific for it. The initial analytical step described in Module 1 (Figure 2A) allows to perform a pre-process analysis of the input files by setting user-defined options for performing adapter cleavage with cutadapt tool [31], as well as quality filtering and analysis of the length distribution of reads. Cutadapt is used for adapter trimming and differs from other adapter trimming tools because it provides several useful option, e.g. error rate assessment in adapter cleavage or search and removal of multiple adapter sequences, essential to get rid of adapter duplications occurring during sequencing library preparation. Module 2 (Figure 2B) allows detection of known miRNAs. To this aim, iMir integrates in its pipeline miRanalyzer stand-alone tool [32], that in its last version (miRanalyzer version 0.3) was improved in speed and features, including a comprehensive analysis of sequences corresponding to isomiR [33]. At this step it is possible to perform also cluster analyses, carrying out PCA analysis and/or applying different hierarchical clustering algorithms. This feature, in fact, is useful when dealing with a very large number of samples to assess similarities and differences among them, such as for example when analyzing results from large cohorts of tumor biopsies. One main advantage of small non-coding RNA sequencing is the possibility to predict novel miRNAs not annotated in databases. This procedure (Module 3, Figure 2C) is performed in iMir with miRanalyzer stand-alone tool [32] and miRDeep2 [34]. With this process it is possible to achieve a dual purpose: (i) to obtain more accurate results on novel miRNAs, that can then be experimentally validated and (ii) to evaluate presence and concentration of reads relative to other sncRNAs in the same datasets. We included in iMir the possibility to implement an intermediate step (Module 4, Figure 2D), before proceeding to differential expression analysis step (Module 5, Figure 2E), to remove the noise and less informative reads, e.g. miRNAs or sncRNAs expressed with very low read counts, that is based on the following statistics: (i) cumulative distribution function and (ii) arbitrary value approaches. Furthermore, low read counts might not reveal a real biological information, being due to sequencing errors or inaccuracy during the procedure of read alignment to the reference genome, such as cross mapping artefacts. To account for this problem, a minimum read count value can be used to filter out reads detected below the cutoff (“Minimum Read Count”, Figure 1B). In addition, after known miRNAs detection (Module 2, Figure 2B), we included statistical approaches to evaluate the cumulative distribution function, such as quartile or percentile values that are computed considering the whole reads-counts to exclude from the list of expressed miRNAs in a given sample those showing read count below that value. This approach can be used also with any sncRNA libraries. The other statistical approach implemented in iMir addresses a common problem encountered when calculating fold-change values (test/control read counts ratios) for RNAs present in the samples at very low levels. Considering a fold-change threshold of ±1.5 and p-value <0.05, when read counts for a given sncRNA are very low this setting can generate biologically irrelevant results. For example, considering a case where 10 tags are assigned to a given RNA in the test sample and 5 in the control, both derived for large cell populations, the resulting fold-change (2.0) may be statistically significant but of doubtful biological relevance [35], contrary to what occurs for sncRNAs showing in the same conditions high read counts. To overcome this problem, we included in iMir the possibility to add in such cases a correction factor. This, computed automatically by the tool as the median of the read count distribution relative to the sncRNA datasets of interest, can be added to the actual read counts of all entries in the datasets. In the case described above, considering a calculated correction factor of 30 the fold-change value calculated for such low-expressed sncRNA will be 1.14, and thus below the threshold set, while for an RNA of the same dataset that shows 4,000 read counts in the sample and 2,000 in the control this adjustment will be irrelevant (see also: Results and discussion). Of note, the user can either disregard this function or use at will an alternative value for this parameter, calculated by any other means desired. These approaches are useful to reduce the number of false-positives detected by differential expression analysis, as these most likely occur among RNAs expressed at a very low level.

Identification and analysis of differentially expressed sncRNAs using digital data is implemented in iMir with two different methods (Module 5, Figures 1C and 2E). The first one, based on the DESeq bioconductor package [36], is particularly suited when biological or technical replicates are available [29]. The second, based on quantile normalization and Fisher’s exact test to assess the statistical relevance, is specially designed for use when no replicates are available [37]. The last iMir module (Module 6, Figure 2F) is designed to perform mRNA targets prediction of expressed, or differentially expressed, miRNAs. mRNA targets are predicted by using miRanda [38,39], that includes current knowledge on target rules and uses a compendium of mammalian miRNAs, and TargetScan [40,41], that computes mRNA targets by searching for the presence of 8mer and 7mer sites matching the seed region of each miRNA. iMir includes in its databases different sncRNAs, such as miRNAs, piRNAs, tRNAs, mRNAs and data from RFam for human, rat and mouse (Additional file 2: Table S1). Performance of iMir was compared with that of the individual bioinformatics tools considered by Williamson et al. [30], selected on the basis of their popularity highlighted by number of citations in the literature. Furthermore, the number of known (available in miRBase) and of novel (absent from the latest release of miRBase) miRNAs detected and the time required to carry to completion the whole analytical flow were evaluated on multiple datasets generated in our laboratory and available from public data repositories and then taken as indicators of iMir performance. The results obtained are in line with what previously reported [30], suggesting reliability of this new tool.

## Results and discussion

**Figure 3:**
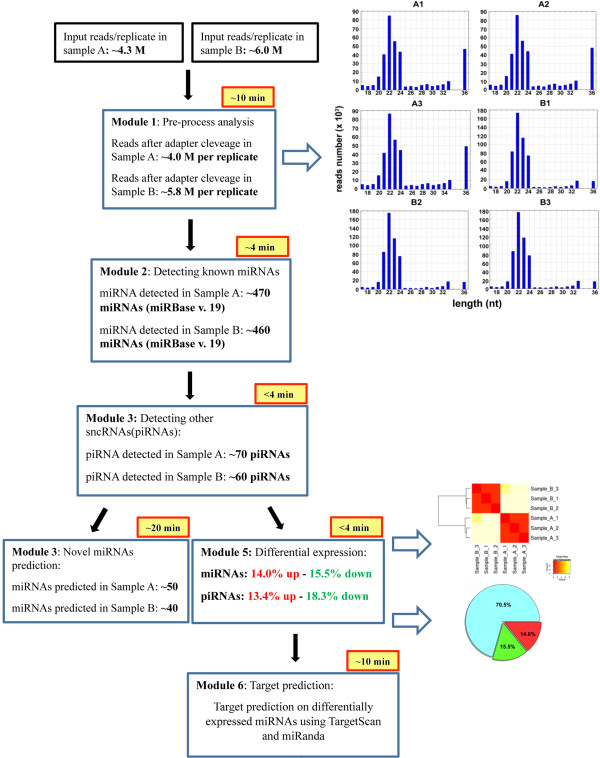
**Graphic representation of iMir pipeline performances.** Datasets obtained from smallRNA-Seq analysis in exponentially growing (sample A) or growth-arrested (sample B) MCF-7 cells, performed in triplicate as described in the text, were input in iMir and analyzed with the standard, complete analytical workflow of the tool. The processing time of each module are highlighted in yellow and the graphic outputs of Modules 1 (histograms showing sequence read length distribution in each replicate) and 5 (heat-map visualization of sncRNA profile differences among samples and pie-chart summarizing the results of the differential expression analysis) are shown to their right.

As an example of the performance of the iMir pipeline, the tool was applied to analyze patterns of sncRNA expression and changes in human breast cancer MCF-7 cells maintained in two different culture conditions affecting cell cycle progression, e.g. growth-arrest and exponential growth [42,43] (see: Additional file 3 for details). For each experimental condition, three sequencing replicates were analyzed to gather a correct estimation also of the technical variability occurring during differential expression analyses. A comprehensive smallRNA-Seq data analysis was performed running all iMir functions with default parameters and the performances of the tool are summarized in Figure 3. To detect classes of sncRNAs other than miRNAs, the raw reads not mapping to known mature miRNAs were aligned against tRNA and mRNA sequences from UCSC Genome Browser, rRNA and piRNA sequences from Nucleotide NCBI database and other sncRNA sequences from RFam [44] (see: Additional file 3 for details). One of the main advantages when applying this procedure is the possibility to reduce false-positive rate in novel miRNAs prediction, while at the same time allowing to search for and analyze other classes of sncRNAs in the datasets. For each sncRNA library sequenced, ~4,0 M raw reads/sample for exponentially growing and ~5.8 M reads/sample for growth arrested cells were obtained (Figure 3 and Table 1). After the pre-process analysis, a small percentage of reads, all <15nt-long, is discarded as the algorithm is unable at present to manage them. The read-length distribution after adapter cleavage in all samples is reported to the right of Module 1 in Figure 3, to show how the majority of reads obtained after this first step are ~22nt long, suggesting that they are mainly due to miRNAs. This observation is further confirmed by the number of reads that actually match known miRNAs (Table 1), computed to account for more than 50% of the entire dataset in each case. The remaining reads, showing a length distribution between 26 and 31nt could include piRNAs, while those 36nt-long could result from longer RNA degradation products. The heatmap reported in Figure 3 to the right of Module 5 highlights the high degree of similarity of miRNA profiles in the three replicates for each sample sequenced (A1-3 for the Case and B1-3 for the Control). A similar results was obtained for piRNAs (data not shown). After that, the iMir module that computes differential expression analysis (Module 5, Figure 2E) was run. miRNA analysis led to the identification of about 460–70 miRNAs per sample (Figure 3 and Table 2), some of which differentially expressed. The pie-charts in Figure 3 summarizes the results of miRNA differential expression analysis performed with DESeq [36], expressed as percentage of detected RNAs showing statistically significant differences in concentration between samples beyond a standard threshold (fold-change ≤ −1.5 or ≥1.5 in exponentially growing *vs* quiescent cells, p-value ≤ 0.05) or not (70.5%). These results, when compared with previously published data relative to miRNA modulation in the same cell line [13,42,43], confirm that iMir is useful to rapidly and efficiently perform differential analysis for these sncRNAs.

**Table 1:** Number of reads before and after adapter cleavage and reads mapped in each sncRNA library included in iMir

**MCF-7 cells**		**Raw reads**	**Reads after adapter Cleavage**	**miRNA reads**	**tRNA reads**	**rRNA reads**	**mRNA reads**	**piRNA reads**	**Remaining reads mapping on the genome**	**Reads not assigned**
Exponentially growing	*Replicate 1*	4,327,501	4,068,141	2,310,200	16,989	91,040	391,750	15,753	597,037	69,516
	*Replicate 2*	4,337,535	4,075,320	2,314,040	17,042	92,178	404,148	16,438	614,614	70,165
	*Replicate 3*	4,354,046	4,091,633	2,374,218	17,737	94,961	420,175	16,949	636,708	71,737
Growth-arrested	*Replicate 1*	6,071,484	5,844,875	4,626,170	13,588	72,460	181,084	14,831	234,955	40,941
	*Replicate 2*	6,075,950	5,846,690	4,621,008	12,470	75,251	185,803	15,122	242,065	40,667
	*Replicate 3*	6,085,784	5,855,090	4,725,975	12,705	77,842	192,161	15,582	249,638	41,494

**Table 2:** Number of known RNAs and of predicted novel miRNAs identified with iMir in replicate smallRNA-Seq datasets from MCF-7 cells

	**Exponentially growing cells**			**Growth-arrested cells**		
	***Replicate 1***	***Replicate 2***	***Replicate 3***	***Replicate 1***	***Replicate 2***	***Replicate 3***
miRNA (miRBase v.20)	473	469	476	461	467	473
tRNA (UCSC Genome Browser)	56	56	54	45	48	47
rRNA (NCBI Nucleotide)	4	4	4	4	4	4
mRNA (RefSeq)	308	307	307	297	320	325
piRNA (NCBI Nucleotide)	86	85	84	73	70	67
Novel miRNA predicted	46	57	55	38	39	42

Recently, an increasing number of studies highlighted the role of piRNAs in breast cancer [45,46]. Since the average length of these RNAs is ~30nt (see: Figure in Additional file 3: Figure S1), smallRNA-Seq represents an efficient analytical approach to assess also absolute and relative expression of these molecules. Based on this assumption, we searched for and analyzed piRNAs in the datasets selected to test iMir performance. To reduce cross-mapping artifacts, reads corresponding to other RNAs, in particular miRNAs, tRNAs, rRNAs and mRNAs, were first filtered out with iMir mapping them against the selected transcribed RNA libraries. This allowed at once to start from a set of more reliable data and to gather information concerning other small RNAs detected by sequencing (Table 1 and Table 2). This analysis led to the identification of 70 and 85 piRNAs expressed in growth-arrested and exponentially growing MCF-7 cells, respectively. Differential piRNA expression analysis and statistical significance testing performed with iMir revealed 12 downregulated and 25 upregulated piRNAs in growing cells, when compared to quiescent ones (p-value = 0.05, threshold = 1.5; Figure 3). We do not have a ready explanation for these relatively low numbers of piRNAs identified in breast cancer cells, except for the fact that piRNAs know to date have been identified in germ cells [21] and it is thus possible that the majority of them is expressed only in these cell types. Furthermore, most piRNAs identified so far associate with the piRNA biogenesis factor Piwil1 (Hiwi) [21,47], that is not detectable in MCF-7 cells, where only Piwil 2 (Hili) and Piwil4 (Hiwi2) are detected [Hashim et al., manuscript in preparation]. As new validated piRNA datasets will become available, for example those identified by association to Piwil 2 and 4, the possibility built into iMir to customize its database will allow to include these in the analysis. The decision to focus here on individual piRNAs instead of considering their genomic organization in clusters is based on the observation that in somatic cells piRNAs deriving from a given cluster show different levels of steady-state expression, possibly due to a specific mechanism of precursor RNA maturation active in these cells or to differences in their half-life. In addition, recent results suggest that individual piRNAs could play important roles in tumor cells [48-50].

We then tested another function of iMir by performing differential miRNAs expression analysis in two different ways: (i) starting directly from the number of raw read-counts obtained with miRanalyzer [32] or (ii) by adding to each of these counts a correction factor (31), computed as the median of the whole read dataset (see above). Once compared, the results obtained with the two approaches showed slight but substantial differences, since ~10% of the miRNAs identified with the first method (pvalue ≤ 0.05) were excluded by the second one (Additional file 4: Table S2). This is explained by the fact that the RNAs expressed at a very low level under both experimental conditions, and thus of uncertain biological significance, were filtered out when using this correction. iMir offers the possibility to choose this method, when needed, also to other classes of sncRNAs.

With respect to the possibility to perform target prediction for selected miRNAs using miRanda and TargetScan databases, another useful function of the tool, it is worth mentioning the possibility for the user to update when required these and the other databases associated to the pipeline, such as those of miRNAs from miRBase, [51-53], of other sncRNAs from different sources and of mRNA targets from TargetScan [40,41,54] and miRanda [38,39].

## Conclusion

We designed, built and describe here iMir, a pipeline that integrates multiple open source modules/resources and implements statistical approaches, combined in an automated flow for high-throughput smallRNA-Seq data analysis. iMir is rapid, accurate and efficient, allowing to examine multiple samples at once and thereby addressing a critical factor for high-throughtput analysis of sncRNA sequencing data, represented by the need for continuous interventions by an operator skilled in informatics and programming. The graphical user interface of iMir, allows a simplified use of the many tools integrated in the pipeline and to customize data analysis according to different needs. In addition, the implementation of different statistical approaches provides the possibility to analyze data according to standard, widely used, as well as to specific needs. Finally, iMir works on Linux and Mac operative systems, user-friendly for biologists with limited skills in informatics. In the future, following the evolution of NGS technologies and recommendations by the scientific community, we plan to keep improving iMir features, including for example tools for sequence variants detection, evolutionary sncRNAs analysis across multiple species and adding specific functions for analysis of emerging classes of small RNAs (pi-, si-, sn-, sno-, ti-RNA, etc.).

## Availability and requirements

**Project name:** iMir.

**Project home page:**http://www.labmedmolge.unisa.it/inglese/research/imir.

**Operating System(s):** Unix/Linux based.

**Other requirements:** Python, Java, Perl, R, DESeq, Bowtie, Vienna RNA Secondary Structure package.

**License:** GNU GPL v3.

**Any restrictions to use by non-academics:** specified by GNU GPL v3.

## Abbreviations

sncRNA: Small non-coding RNA; miRNA: microRNA; NGS: Next generation sequencing.

## Competing interests

The authors declare that they have no competing interests.

## Authors’ contributions

GG and MRDF lead in the conception and design of the software, performed the statistical analyses and coordinated manuscript drafting and revision. AR participated in conception and development of the software. AH and FR contributed to software conception and design and to manuscript drafting. GN, MR and RT carried out smallRNA-Seq analyses, manuscript drafting and contributed to software conception. AW coordinated the project, participated in conception of the software and participated in drafting and finalization of the manuscript. All authors read and approved the final manuscript.

## Supplementary Material

Additional file 1 User Manual. Click here for file

Additional file 2: Table S1 Summary of annotated small non-coding RNAs included in iMir database. Click here for file

Additional file 3 Additional Materials and Methods and Additional Figure Legend. Click here for file

Additional file 4: Table S2 List of miRNAs differentially expressed in exponentially growing *vs* growth-arrested MCF-7 human breast cancer cells (raw and adjusted read-counts, with associated fold-changes and p-values). Click here for file
