# Supplementary material for: iMir: An integrated pipeline for high-throughput analysis of small non-coding RNA data obtained by smallRNA-Seq
Source: BMC Bioinformatics. 2013 Dec 13;14:362. doi: 10.1186/1471-2105-14-362 (PMC3878829; doi:10.1186/1471-2105-14-362)
Supplement: Additional file 1 — User Manual. [file 1471-2105-14-362-S1.pdf]

iMir User Manual

Integrated pipeline for HT miRNA-Seq data analysis

Laboratorio di Medicina Molecolare e Genomica
Università degli Studi di Salerno
Via S. Allende, 1
84081 Baronissi (Sa)
http://www.labmedmolge.unisa.it

Index

1. How to use iMir 5

2. Installation procedure on Linux (Ubuntu) 12

2.1 Prerequisites 12

2.1.1 make, g++ and gcc 12

2.1.2 Python and Perl 12

2.1.3 Java 12

2.1.4 R 12

2.2 Dependences 12

2.2.1 Linux packages 12

2.2.2 Python libraries 12

2.2.3 Bowtie 13

2.2.4 Cutadapt 13

2.2.5 Vienna RNA Secondary Structure package 13

2.2.6 Weka 13

2.2.7 DESeq 13

2.2.8 gplots 13

2.2.9 limma 14

2.2.10 SQUID (for miRDeep2) 14

2.2.11 randfold (for miRDeep2) 14

2.2.12 PDF::API2 (for miRDeep2) 14

2.3 Installation procedure 14

2.3.1 g++ and gcc installation 14

2.3.2 make installation 15

2.3.3 numpy installation 15

2.3.4 matplotlib installation 15

2.3.5 rpy2 installation 15

2.3.6 Tkinter installation 16

2.3.7 Vienna RNA Secondary Structure package installation 16

2.3.8 cutadapt installation 16
2.3.9 DESeq Bioconductor package 16
2.3.10 gplots package 16
2.3.11 limma package 16
2.3.12 miRDeep2 installation 17
3. Installation procedure on MacOS 18
3.1 Prerequisites 18
3.1.1 make, g++ and gcc 18
3.1.2 Python and Perl 18
3.1.3 Java 18
3.1.4 R 18
3.2 Dependences 19
3.2.1 Bowtie 19
3.2.2 Vienna RNA Secondary Structure Package 19
3.2.3 Cutadapt 19
3.2.4 Weka 19
3.2.5 DESeq 19
3.2.6 gplots 19
3.2.7 limma 19
3.2.8 SQUID (for miRDeep2) 20
3.2.9 randfold (for miRDeep2) 20
3.2.10 PDF::API2 (for miRDeep2) 20
3.3 Installation procedure 20
3.3.1 numpy installation 20
3.3.2 matplotlib installation 21
3.3.3 rpy2 installation 21
3.3.4 cutadapt installation 21
3.3.5 Vienna RNA Secondary Structure package installation 21
3.3.6 DESeq Bioconductor 22

3.3.9 miRDeep2 installation 22

4. Database 24

4.1 iMir General structure of the database 24

4.2 Database population 24

5. Output 25

1. How to use iMir

iMir scripts can be launched directly using PYTHON after dependencies have been installed and "iMir_DB" folder of the species of interest have been downloaded (for more details see section 4 of this documentation). After installing prerequisites, iMir can be installed using the script `install_iMir.sh` included in iMir folder. In this case the dependences software will be automatically installed.

To run *iMir*, change directory containing the *iMir.py* file and run the script:

```
> cd [path of iMir_folder](cd ../iMir/src/)
> python iMir.py #to launch iMir Graphical User Interface
```

The ">" (or "$") indicates command prompt and should not be typed.

Squared brackets indicates text that needs to be adjusted. Brackets should not be typed. Comments are indicated by "#".

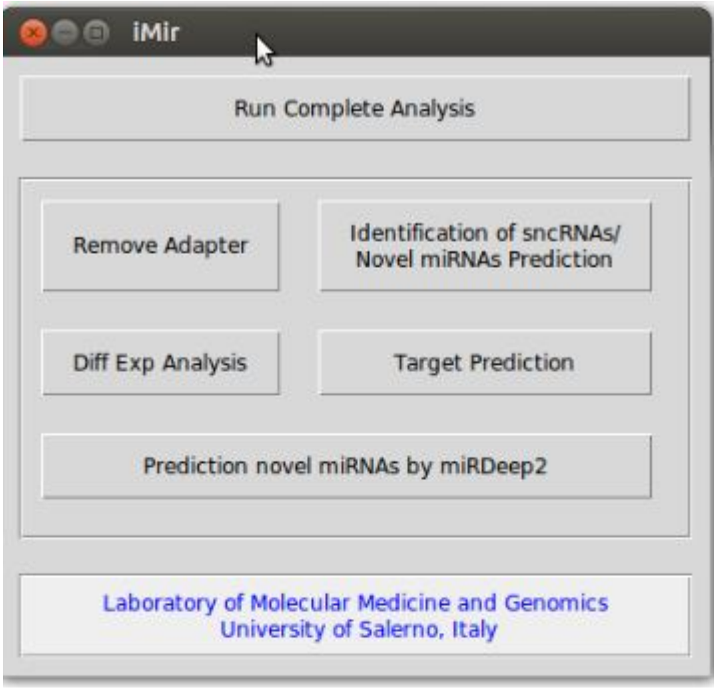

Figure 1 *iMir* main menu

After launched the command line, the iMir menu (Figure 1) is displayed. This allows to select the preferred computational step:

- **Run Complete Analysis**: performs a complete analysis starting from raw NGS data.
- **Remove Adapter**: removes the adapters sequences (3' and/or 5'), performs a quality filter on adapters sequences and set a read-count cutoff starting from fastq files.

- **Identification of sncRNAs /Novel miRNAs Prediction**: identification of known small non coding RNA (sncRNAs) and novel miRNAs prediction. The input must be given in reads-count format ("read sequence"<tab> "read frequency").

- **Target Prediction**: given a list of miRNAs, allows the identification of mRNA targets. The miRNA name must be written in inverted commas (“ ”).

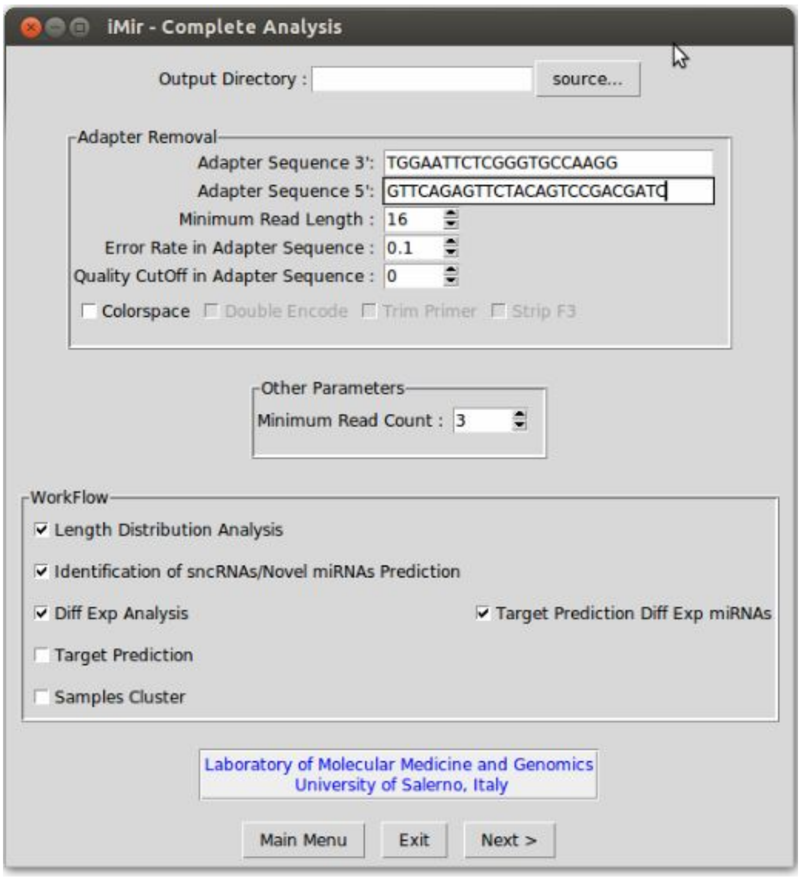

Figure 2 Complete-Analysis menu 1

When **Run Complete Analysis** is selected *iMir* GUI window shown in Figure 2 is displayed.

This menu allows:

- To select the output folder.
- To set parameters for pre-process analysis (*Adapter Removal* and *Other Parameters sections*) such as:
    - **3’ adapter sequence**
    - **5’ adapter sequence**
    - **Minimum sequence length to consider after adapter trimming**

- **Percentage of error-rate in adapter sequence**
- **Quality cutoff in adapter sequence**
- **Minimum read-count cutoff**

- To define the analytical steps to perform (**WorkFlow** *section*) on NGS raw data:
  - **Length Distribution Analysis**: removes adapter sequences according the parameters define above and produces histograms about read-length distribution.
  - **Identification of sncRNAs / Novel miRNAs prediction:** enables the module to predict known sncRNAs and predict novel miRNAs.
  - **Diff Exp analysis:** enables the module to perform differential expression analysis.
  - **Target prediction on Diff Exp miRNAs** (when *Diff Exp* is selected)**:** allows to predict mRNA target based on the list of differentially expressed miRNAs.
  - **Target prediction:** enables the module to predict mRNA targets for all miRNAs detected.
  - **Sample Cluster/PCA Anlysis:** enables module to perform cluster analysis.

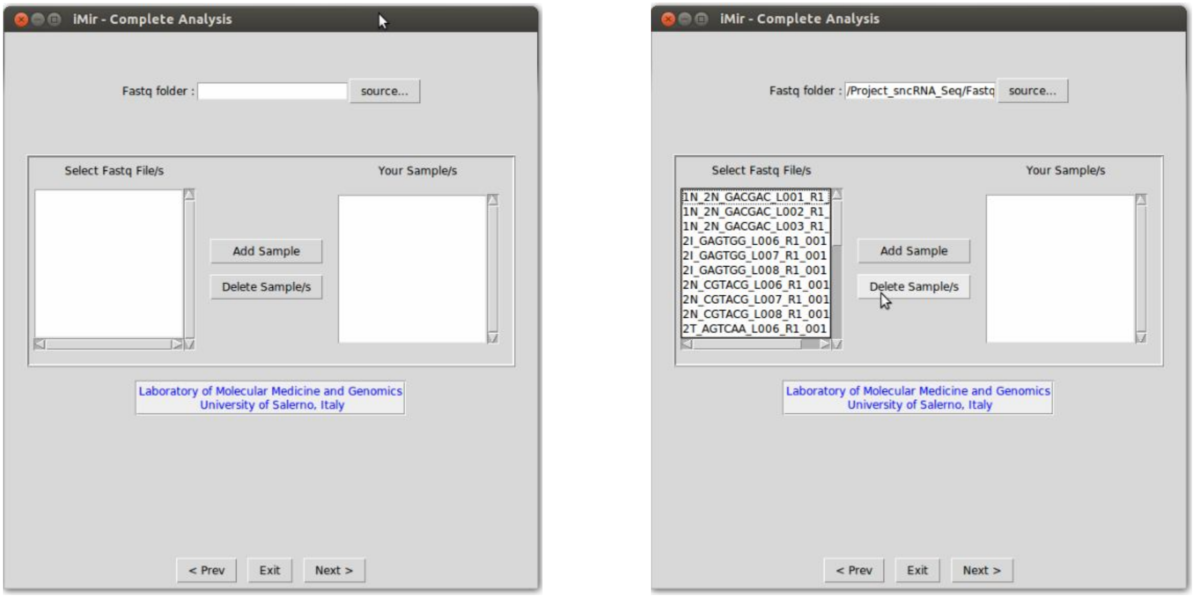

Figure 3 Samples input windows.

After setting the initial parameters (Figure 2), the next step is to select the input files. Clicking on **[*source...*].** you select input folder that will display the corresponding fastq files on the left part of the window. (Figure 3).

To load the sample select the file/s and click on **Add Sample** and type the corresponding name (or group name). It will be displayed on the right panel (Figure 3).

The screen-shot displayed in Figure 4 represents the main window of *iMir GUI*. It is divided in several sections, according the analytical step to perform.

- ***Mandatory Parameters and Optional Parameters***: These sections allows to define miRanalyzer parameters. (For details about miRanalyzer parameters visit the web link *://bioinfo5.ugr.es/miRanalyzer/standalone.html#x1-100004*).
    - *Mandatory Parameters*: In this section is possible to specify:
        - *Species* of interest. A menu-bar allows to select amongs "Human", "Rat" and "Mouse" species, filling automatically the "Basename of bowtie index" and "Species Short" fields. If the smallRNA-Seq experiment is performed on an another species, not included in the menu bar, is possible to fill manually the "Basename of bowtie index" and "Species Short" fields, after populating iMir_DB with the track corresponding to the species of interest.
            - *Basename of bowtie index*: Assembly/Basename of bowtie index, i.e. hg19,mm9, etc.
            - *Species Short*: 'short name of species': This must be the abbreviation of the species used in miRBase for example hsa for Homo sapiens or mmu for Mus musculus.
        - *Kingdom*: In order to set the models and features for the prediction of new microRNAs the program needs to know whether the species is a plant or animal.
        - *dbPath:* the absolute/complete path to the miRanalyzer database (for Human i.e. `../iMir_DB/Human/miRanalyzerDB`)
        - *bowtiePath*: the path with the Bowtie binaries (i.e. `/usr/bin`).
    - *Optional Parameter*: In This section is possible to specify the other miRanalyzer parameters (For details about miRanalyzer parameters visit the web link *://bioinfo5.ugr.es/miRanalyzer/standalone.html#x1-100004*).
        - If *justKnown* check-box and *justNew* check-box are both not selected a complete analysis will be performed, to detect known miRNAs, other sncRNA expressed in the sample of interest and predict novel miRNAs.
        - *justKnown*: Clicking on this check-box the analysis will be performed to detect only known miRNAs.
        - *justNew*: Clicking on this check-box the analysis will be performed to predict novel miRNAs.
        - *Transcribe Libreries*: In this section is possible to specify the other class of sncRNAs to investigate. Clicking on *Forward* (or *Reverse*) check-box will attivate

the menu-bar *Add* to include the class of sncRNA to study. *Delete* option alloes to exclude that specific class.

        - *Percentile Filter*: This parameter allows to specify a value between 0-100 to specify the percentile to use to filter out sncRNA with low read-counts.

- *Diff Exp Parameters*: This section allows to define the samples and the parameters to use in differential expression analysis.
    - *Correction factor*: This parameter allows to select a value to add to each count in the whole read dataset. By default, a correction factor of 1 is added to each read-count of the sncRNA dataset. This value can be set by user (*Defined by User parameter*) or computed as median value (*Median Value parameter*) of the whole read-counts dataset of test *vs* control sample. Set *Defined by User parameter* to 0 if no value must add.
    - *Diff Exp Process*: This radio button allows to select which method use for differential expression analysis. If *DESeq* is selected *Fit Type* radio button will be activate to select the Fit Type method used by DESeq.
    - *Select Test* (*Select Control*) subsection: Just clicking once on the samples name that appear in *Select Test* and *Select Control* columns and then on ">>" will be possible to define the sample couple on which carry out differential expression analysis.
    - *p-value* and *Adj-pVal*: These radio button allows to specify if consider p-value or adjusted p-value for differential expression analysis. It is also possible to define the p-value cutoff.
    - *Fold-Change Threshold*: In this field is possible to define the Fold-Change cutoff to use for differential expression analysis.
- ***Target prediction section***: Clicking once on sample name displayed in the window is possible to define the sample on which perform mRNA target prediction. Clicking on *Select All* radio button is possible to select all the samples.
- ***Cluster Analysis section***: Clicking once on sample name displayed in the window is possible to select samples on which perform hierarchical cluster analysis.
- ***Terminal****:* This window allows to follow the analysis flow, once pressed *Start Analysis* button.

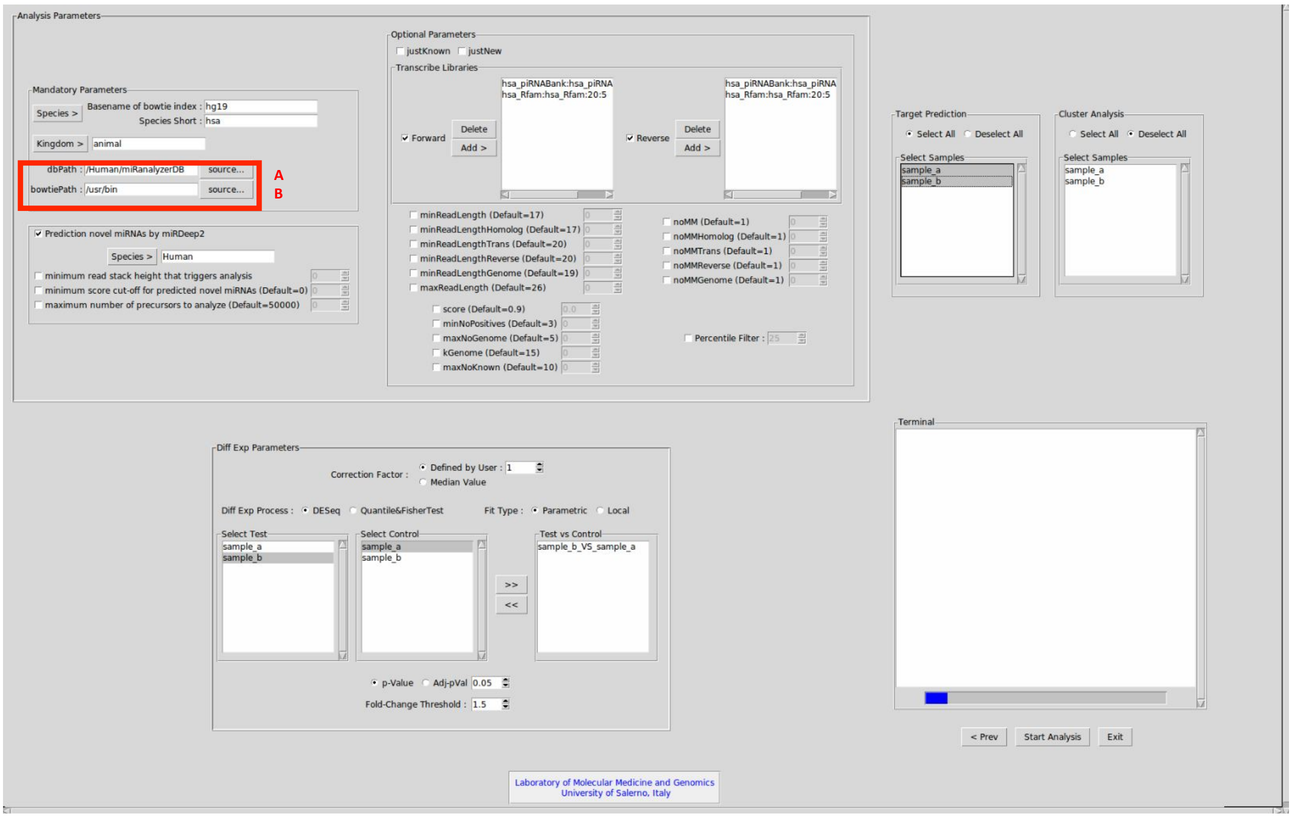

Figure 4 *iMir* Main window. The red rectangle highlight how provide (A) the path to the Bowtie indices and (B) the path to the Bowtie binaries.

Clicking on **Remove Adapter** button in iMir main menu (Figure 1) a new window (Figure 5) is displayed. It allows to insert the parameters need for removing the adapter sequences from the fastq files.

The **Diff Exp Analysis module** button in iMir main menu (Figure 1) allows to run differential expression analysis starting from a tab-delimitated input file containing the read-counts for control and test samples respectively (Figure 6 left). By clicking "**Next**" a new window will open. Here you should declare the sample’s name and the number of replicates.

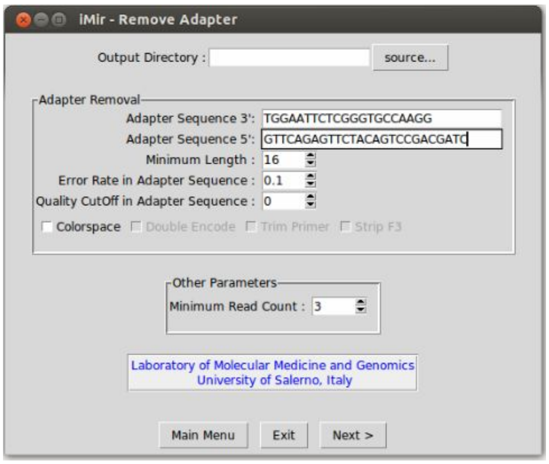

Figure 5 *iMir* Remove Adapter window

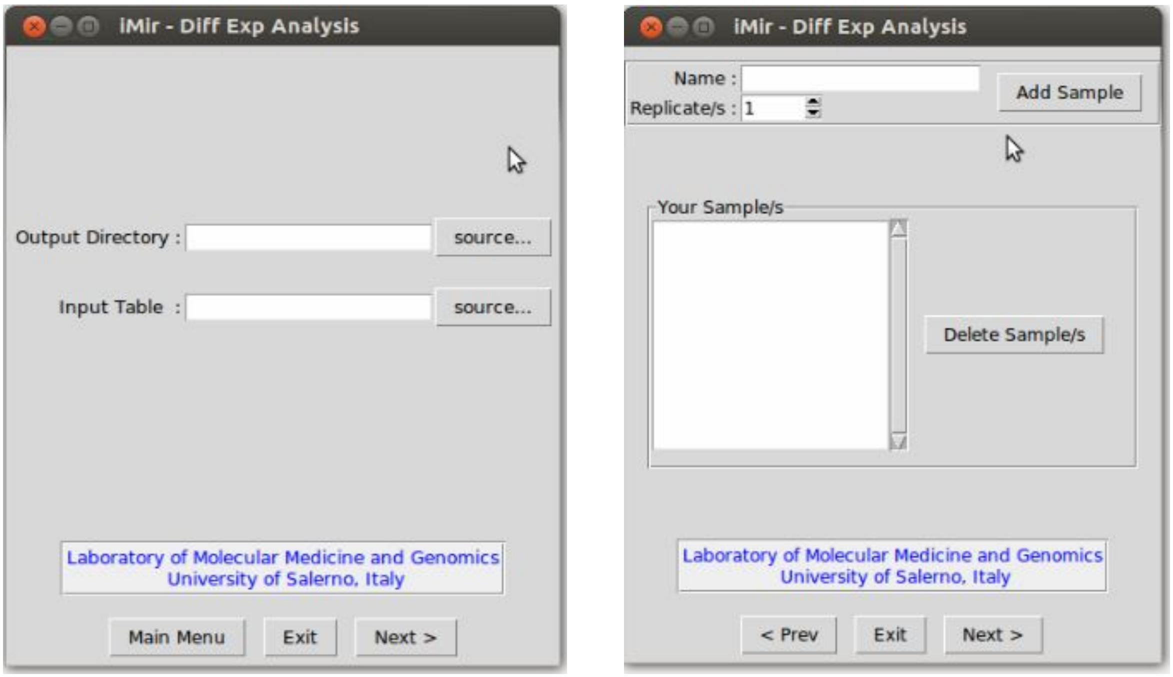

Figure 6 *iMir* Diff Exp Analysis window

The **Target Prediction** module (Figure 1) allows to perform a mRNA target prediction for a list of miRNAs. The miRNA name must be written in inverted commas, like “has-miR-10”.

2. Installation procedure on Linux (Ubuntu)

Prerequisites software must be installed before to run `install_iMir.sh` script. The dependencies software will be installed automatically by `install_iMir.sh` script.

2.1 Prerequisites

2.1.1 make, g++ and gcc

To run the installation script is mandatory that the following packages have been installed on own system:

- gcc
- g++
- make

2.1.2 Python and Perl

Most operating systems have built-in PYTHON and PERL and no installation is required. If that is not the case, PYTHON can be downloaded from http://python.org/getit/ and PERL from http://www.perl.org/get.html.

Python version 2.7

Perl version 5.0

2.1.3 Java

Most operating systems have built-in JAVA and no installation is required. If that is not the case, JAVA can be downloaded from http://www.java.com/en/download/.

Java version ≥ 1.6

2.1.4 R

R is required to perform differential expression analysis with DESeq Bioconductor package. It can be found at http://mirror.fcaglp.unlp.edu.ar/CRAN/.

R version ≥3.0.1.

2.2 Dependences

2.2.1 Linux packages

The following Linux packages are needed to run iMir:

- python-dev (required by cutadapt tool)
- libxml2 and libxml2-dev (required to install R DESeq Bioconductor)
- libfreetype6 and libfreetype6-dev (required to install matplotlib PYTHON library)

2.2.2 Python libraries

- numPy

- matplotlib
- rpy2
- Tkinter

2.2.3 Bowtie

Bowtie is required by miRanalyzer stand-alone tool and miRDeep2. It should be installed and added to the system path, so that typing bowtie in the terminal activates the program regardless of the working directory (e. g typing as root `'ln -s path/to/Bowtie_folder/bowtie /usr/bin/bowtie'`). It can be found at http://sourceforge.net/projects/bowtie-bio/files/bowtie/0.12.8/.

2.2.4 Cutadapt

Cutadapt is required to remove adapter sequences from high-throughput sequencing data. It should installed and can be downloaded from http://code.google.com/p/cutadapt/. Before to proceed with installation could be necessary to install python-dev library.

2.2.5 Vienna RNA Secondary Structure package

Vienna RNA Secondary Structure package is required by miRanalyzer stand-alone tool. iMir currently works with 1.8.5 or earlier versions. It can be downloaded from web site http://www.tbi.univie.ac.at/~ivo/RNA/.

2.2.6 Weka

Weka is required by miRanalyzer. It is a data mining software in JAVA. The tested version is weka 3.5.3. "weka.jar" file, (named like this, without version) must be in the some folder as miRanalyzer.jar. In iMir folder it is yet included (inside miRanalyzer subfolder).

2.2.7 DESeq

DESeq is a R package to analyse count data from Next-Generation-Sequencing experiment and test for differential expression. It is used by iMir when test/control samples are inserted in *DiffExp_Samples* section of Sample Sheet. DESeq version >= 1.9

It can be found at http://www.bioconductor.org/packages/2.11/bioc/html/DESeq.html .

Sometime before to install DESeq could be necessary to install libxml2-dev library.

2.2.8 gplots

gplots R package is required to create plots during differential expression analysis and cluster analysis. It can be downloaded from http://cran.r-project.org/web/packages/gplots/index.html .

2.2.9 limma

Limma R package is required to perform quantile normalization for differential expression analysis. It can be downloaded from http://www.bioconductor.org/packages/2.11/bioc/html/limma.html .

2.2.10 SQUID (for miRDeep2)

SQUID library is required by miRDeep2. It can be downloaded from http://selab.janelia.org/software.html.

2.2.11 randfold (for miRDeep2)

randfold is required by miRDeep2. It can be downloaded from http://bioinformatics.psb.ugent.be/details/Randfold

2.2.12 PDF::API2 (for miRDeep2)

The Perl package PDF::API2 is required by miRDeep2. It can be downloaded from http://search.cpan.org/search?query=PDF%3A%3AAPI2&mode=all

2.3 Installation procedure

The *iMir* pipeline can only be installed in a Linux/UNIX-like operating system (Linux/UNIX or MAC OS X). Most of the dependencies can be automatically installed during the iMir installation using the installation script. Only R have to be manually installed on the system.

To install *iMir* and/or all dependencies move on *iMir* directory (`iMir/src/`). Then simply use the following command (with administration rights - > `sudo su`):

```
> cd ../iMir/src/
```

```
> sh install_iMir.sh
```

Follow the different steps of the installation and answer the question ask by the program.

Change access to miRDeep2 folder typing:

```
> sudo chmod -R 775 .../iMir/src/miRDeep2
```

When installation procedure is finished is necessary reboot the system.

Without the install *iMir* script follow the instructions given below

2.3.1 g++ and gcc installation

Under Linux-Ubuntu system,with administration rights, open a command shell and type:

```
>sudo apt-get install g++
```

2.3.2 make installation

Under Linux-Ubuntu system, with administration rights, open a command shell and type:

```
>sudo apt-get install build-essential
```

2.3.3 *numpy* installation

After downloaded, unpack and compile it.

```
>tar xvzf numpy.xx.xx.tar.gz
>cd [path of numpy folder]
>python setup.py build
```

(as root) `>python setup.py install`

To check the installation launch Python and type:

```
import numpy
numpy
```

You should recognize the installation path.

2.3.4 *matplotlib* installation

After downloaded it, unpack and compile it.

```
>tar xvzf matplotlib.tar.gz
>cd [path of matplotlib folder]
>python setup.py build
```

(as root) `>python setup.py install`

To check the installation launch Python and type

```
import matplotlib
matplotlib
```

You should recognize the installation path.

2.3.5 *rpy2* installation

After downloaded it, unpack and compile it.

```
>tar xvzf rpy2.tar.gz
>cd [path of rpy2 folder]
>python setup.py build
```

(as root) `>python setup.py install`

To check the installation launch Python and type

```
import rpy2
```

rpy2

You should recognize the installation path.

2.3.6 Tkinter installation

Under Linux Ubuntu system, with administration rights, open a command shell and type:

```
>sudo apt-get install python-tk
```

2.3.7 Vienna RNA Secondary Structure package installation

After downloaded it, unpack it

```
>tar xvzf ViennaRNA-1.8.xx.tar.gz
>cd [path of Vienna folder]
>./configure
>make
```

(as root) `>make install`

2.3.8 cutadapt installation

After downloaded it, unpack it

```
>tar xvzf cutadapt-1.2.1.tar.gz
>cd [path of cutadapt folder]
>python setup.py build
>python setup.py install
```

2.3.9 DESeq Bioconductor package

Start R and enter:

```
source("http://bioconductor.org/biocLite.R")
biocLite(BioUpdate)
biocLite("DESeq")
```

2.3.10 gplots package

Start R and enter:

```
install.packages(“gplots”)
```

2.3.11 limma package

Start R and enter:

```
source("http://bioconductor.org/biocLite.R")
biocLite(BioUpdate)
biocLite("DESeq")
```

2.3.12 miRDeep2 installation

After dowloaded all necesary packages described in 2.2 Dependences:

- attach the miRDeep2 executable path to your PATH typing(as root):
  `>echo 'export PATH=$PATH:path_to_miRDeep2_in_iMir_folder' >>/etc/profile`
- install SQUID library typing (as root):
  ```
  >tar xxvzf squid-1.9g.tar.gz
  >cd SQUID
  >./configure
  >make
  > make install
  ```
- install randfold library typing (as root):
  ```
  > tar xvvzf randfold-2.0
  > cd randfold
  ```
  edit makefile changing line with `INCLUDE=-I.` to `INCLUDE=-I. -Ipath_to_squid -Lpath_to_squid`
  ```
  >make
  ```
  add randfold to your PATH variable:
  ```
  >echo 'export PATH=$PATH:path_to_randfold' >> /etc/profile
  ```
- install PDF::API2 (as root):
  Before to install PDF::API2 could be necessary install the perl library FONT::TTF.
  Under Linux Ubuntu system can be installed typing:
  ```
  >sudo apt-get install libfont-ttf-perl
  ```
  PDF::API2 can be installed after downloaded the library typing:
  ```
  > tar xvvzf PDF-API-x.xx.tar.gz
  ```
  `>cd` to you PDF_API2 directory
  ```
  >perl Makefile.PL PREFIX=path_to_mirDeep2_in_iMir_folder
  LIB=path_to_miRDeep2_in_iMir_folder/lib
  >make
  >make test
  >make install
  > echo 'export PERL5LIB=PERL5LIB:path_to_miRDeep2_in_iMir_folder/lib/perl5/5.x/'
  >> /etc/profile
  ```
- Change access to miRDeep2 folder typing:
  ```
  > sudo chmod -R 775 miRDeep2
  ```
- Restart the System

3. Installation procedure on MacOS

Prerequisites software must be installed before to run `install_iMir_MAC.sh` script. The dependencies software will be installed automatically by `install_iMir_MAC.sh` script.

3.1 Prerequisites

3.1.1 make, g++ and gcc

To run the installation script is mandatory that the following packages have been installed on own system:

- gcc
- g++
- make

These packages can be installed using Xcode

3.1.2 Python and Perl

MacOS have built-in PYTHON and PERL and no installation is required. If that is not the case or is necessary to update them, PYTHON can be downloaded from http://ww.python.org/getit/ and PERL from http://www.perl.org/get.html. In addition could be necessary install GNU Fortran Compiler. It can be downloaded from http://r.research.att.com/tools/.

Python version 2.7

Perl version 5

PYTHON libraries:

numpy (http://www.scipy.org/Installing_SciPy/Mac_OS_X);

matplotlib (http://sourceforge.net/projects/matplotlib/files/matplotlib/matplotlib-1.1.1/);

rpy2 (http://sourceforge.net/projects/rpy/files/OldFiles/).

3.1.3 Java

Most operating systems have built-in JAVA and no installation is required. If that is not the case, JAVA can be downloaded from http://www.java.com/en/download/.

3.1.4 R

R is required to perform differential expression analysis with DESeq Bioconductor package. It can be found at http://mirror.fcaglp.unlp.edu.ar/CRAN/. R version >=3.0.1.

3.2 Dependences

3.2.1 Bowtie

Bowtie is required by miRanalyzer stand-alone tool. It should be installed and add to system path, so that typing bowtie in the terminal activates the program regardless of the working directory (e.g. typing as root `'ln -s path/to/Bowtie_folder/bowtie /usr/bin/bowtie'`). It can be found at http://sourceforge.net/projects/bowtie-bio/files/bowtie/0.12.8/.

3.2.2 Vienna RNA Secondary Structure Package

Vienna RNA Secondary Structure package is required by miRanalyzer stand-alone tool. iMir currently works with 1.8.5 or earlier versions. It can be downloaded from web site http://www.tbi.univie.ac.at/~ivo/RNA/.

3.2.3 Cutadapt

Cutadapt is required to remove adapter sequences from high-throughput sequencing data. It should installed and can be downloaded from http://code.google.com/p/cutadapt/. Before to proceed with installation could be necessary to install python-dev library.

3.2.4 Weka

Weka is required by miRanalyzer. It is a data mining software in JAVA. The tested version is weka 3.5.3. "weka.jar" file, (named like this, without version) must be in the same folder as miRanalyzer.jar. In iMir folder it is yet included (inside miRanalyzer subfolder).

3.2.5 DESeq

DESeq is a R package to analyse count data from Next-Generation-Sequencing experiment and test for differential expression. It is used by iMir when case/control samples are inserted in *DiffExp_Samples* section of Sample Sheet. It can be found at http://www.bioconductor.org/packages/2.11/bioc/html/DESeq.html.

3.2.6 gplots

gplots R package is required to create plots during differential expression analysis and cluster analysis. It can be downloaded from http://cran.r-project.org/web/packages/gplots/index.html.

3.2.7 limma

Limma R package is required to perform quantile normalization for differential expression analysis. It can be downloaded from http://www.bioconductor.org/packages/2.11/bioc/html/limma.html .

3.2.8 SQUID (for miRDeep2)

SQUID library is required by miRDeep2. It can be downloaded from http://selab.janelia.org/software.html.

3.2.9 randfold (for miRDeep2)

randfold is required by miRDeep2. It can be downloaded from
http://bioinformatics.psb.ugent.be/details/Randfold

3.2.10 PDF::API2 (for miRDeep2)

The Perl package PDF::API2 is required by miRDeep2. It can be downloaded from
http://search.cpan.org/search?query=PDF%3A%3AAPI2&mode=all

3.3 Installation procedure

The iMir pipeline can only be installed in a Linux/UNIX-like operating system (Linux/UNIX or MAC OS X). Most of the dependencies can be automatically installed during the iMir installation using the installation script. Only R have to be manually installed on the system.

To install iMir and/or all dependencies move on iMir directory (`iMir/src/`). Then simply use the following command (with administration rights > `sudo su`):

```
> cd ../iMir/src/

>sh install_iMir_MAC.sh
```

Follow the different steps of the installation and answer the question ask by the program.

Change access to miRDeep2 folder typing:

```
> sudo chmod -R 775 .../iMir/src/miRDeep2
```

When installation procedure is finished is <u>necessary reboot the system</u>.

Without the install iMir script follow the instructions given below

3.3.1 *numpy* installation

After downloaded, unpack and compile it.

```
>tar xvzf numpy.xx.xx.tar.gz
>cd [path of numpy folder]
>python setup.py build
```

(as root) `>python setup.py install`

To check the installation launch Python and type:

```
import numpy
numpy
```

You should recognize the installation path.

3.3.2 *matplotlib* installation

After downloaded it, unpack and compile it.

```
>tar xvzf matplotlib.tar.gz
>cd [path of matplotlib folder]
>python setup.py build
```

(as root) `>python setup.py install`

To check the installation launch Python and type

```
import matplotlib
matplotlib
```

You should recognize the installation path.

3.3.3 rpy2 installation

After downloaded it, unpack and compile it.

```
>tar xvzf rpy2.tar.gz
>cd [path of rpy2 folder]
>python setup.py build
```

(as root) `>python setup.py install`

To check the installation launch Python and type

```
import rpy2
rpy2
```

You should recognize the installation path.

3.3.4 cutadapt installation

After downloaded it, unpack it

```
>tar xvzf cutadapt-1.2.1.tar.gz
>cd [path of cutadapt folder]
>python setup.py build
>python setup.py install
```

3.3.5 Vienna RNA Secondary Structure package installation

After downloaded it, unpack it

```
>tar xvzf ViennaRNA-1.8.xx.tar.gz
```

```
>cd [path of Vienna folder]
>./configure
>make
```

(as root) `>make install`

3.3.6 DESeq Bioconductor

Start R and enter:

```
source("http://bioconductor.org/biocLite.R")
biocLite("DESeq")
```

3.3.7 gplots package

Start R and enter:

```
install.packages(“gplots”)
```

3.3.8 limma package

Start R and enter:

```
source("http://bioconductor.org/biocLite.R")
biocLite(BioUpdate)
```

3.3.9 miRDeep2 installation

After dowloaded all necesary packages described in 3.2 Dependences:

- attach the miRDeep2 executable path to your PATH typing(as root):
  `>echo 'export PATH=$PATH:path_to_miRDeep2_in_iMir_folder' >>/etc/profile`
- install SQUID library typing (as root):
  ```
  >tar xxvzf squid-1.9g.tar.gz
  >cd SQUID
  >./configure
  >make
  > make install
  ```
- install randfold library typing (as root):
  ```
  > tar xvvzf randfold-2.0
  > cd randfold
  ```
  edit makefile changing line with `INCLUDE=-I.` to `INCLUDE=-I. -Ipath_to_squid -Lpath_to_squid`
  `>make`
  add randfold to your PATH variable:
  `>echo 'export PATH=$PATH:path_to_randfold' >> /etc/profile`
- install PDF::API2 (as root):

Before to install PDF::API2 could be necessary install the perl library FONT::TTF(You find PDF::API2 package inside miRDeep2/essential).
Under MacOS system can be installed typing:

```
> tar xvvzf FONT-TTF-x.xx.tar.gz
>cd to you  FONT-TTF directory
>perl Makefile.PL
>make
>make test
>make install
```

(You find PDF::API2 package inside miRDeep2/essential):

```
> tar xvvzf PDF-API-x.xx.tar.gz
>cd to your PDF_API2 directory
>perl Makefile.PL PREFIX=path_to_mirDeep2_in_iMir_folder
LIB=path_to_miRDeep2_in_iMir_folder/lib
>make
>make test
>make install
> echo 'export PERL5LIB=PERL5LIB:path_to_miRDeep2_in_iMir_folder/lib/perl5/5.x/'
>> /etc/profile
```

- Change access to miRDeep2 folder typing:

```
> sudo chmod -R 775 miRDeep2
```

- Restart the System

4. Database

4.1 iMir General structure of the database

In iMir the huge number of libraries, like mature microRNA, precursor microRNA, chromosome sequences, bowtie indexes, etc., are stored in a local file-base database called "iMir_DB". Inside the base directory there is a subdirectory, "miRanalyzerDB" used by miRanalyzer stand-alone version.

A full description of the database used by miRanalyzer can be found at miRanalyzer web-page, section "General Structure of the database"
(*http://bioinfo5.ugr.es/miRanalyzer/standalone.html#x1-50003.1*).

The miRanalyzerDB contains the folders:

- bowtie: the folder where the bowtie indexes must be.
- model: this folder holds the model files for the prediction of the new microRNA
- out: the default output folder, a folder with the name of the input file will be generated in this directory.
- seqOBJ: the genome sequences in miRanalyzer format.

The bowtie folder is divided in several subfolders which need to be name exactly as follows:

- genome: the indexes of the whole genome sequences. The name must be the same as used for the genome sequences in seqOBJ folder.
- mature: the indexes for the mature microRNA
- hairpin: the indexes for the hairpin - precursor- sequences of microRNAs
- translibs: the indexes of the other libraries which should be used. It is a customize library. The files can be generated with bowtie-built.

4.2 Database population

iMir provides the libraries to analyze miRNA-Seq experiments from human, mouse, and rat. For each species of interest is possible to download the corresponding iMir_DB folder, organized as described above. iMir refers to miRBase v.20 for the mature and hairpin sequences and to RFam v11.0 (taken from http://rfam.sanger.ac.uk/), piRNA (for human, rat and mouse from Nucleotide NCBI and for human and rat from PiRNABank). RefSeq (from http://genome.ucsc.edu/) and tRNA(from http://genome.ucsc.edu/) for translibs sequences.

5. Output

After the tool has finished the analysis, an output folder is created in the user-specific location. The output folder includes:

- *Sample_name*.fa: A fasta file in which each sequence only occurs once. To indicate how many reads the sequence represents is added a suffix to each fasta identifier. E.g.: a sequence representing one hundred reads in the data will have the 'x_100' suffix in the identifier.
- *Sample_name*_converted.rc: A tab delimitated file, containing the unique reads and their counts.
- *Sample_name*_LengthDistribution.png: Histogram describing read length distribution.
- *Sample_name*_LengthDistribution.txt: Text file containing values used to generate the LengthDistrivution histogram .
- *Sample_name*_sncRNA_result folder: Results of miRanalyzer analysis. It contains several files and folders. A detailed explanation about output files can be found on miRanalyzer website - download section [http://bioinfo5.ugr.es/miRanalyzer/standalone.html#toc-Section-5]
- Cluster_Table_Raw_Data.txt: a tab delimitated file with all miRNAs expressed in all samples and the corresponding read_count values
- DESeq_Analysis folder: Results of differential expression analysis. It produces several folders:
  - *Case_Sample_Name*_vs_*Control_Sample_Name* folder, including:
    - *Case_Sample_Name*_vs_*Control_Sample_Name*.dat: Input file for DESeq package. It is a tab-delimitated table containing all miRNAs expressed in *Case_Sample* and *Control_Sample* and corresponding read-count values.
    - DESeq_*Case_Sample_Name*_vs_*Control_Sample_Name*.txt: Output file of DESeq analysis. A detailed explanation of DESeq output can be found on DESeq documentation
    - miRDiffList_for_Target.txt: A text file containing the list of miRNAs whereby perform target prediction.
    - Result_Conversion_20_15.txt: A text file containing results of miRNAs name conversion from miRBase v.20 to v.15.

- Result_Conversion_20_17.txt: A text file containing results of miRNas name conversion from miRBase v.20 to v.17.
- Miranda Folder: Target prediction results performed on microRNA.org. The folder is divided in two subfolder *Conserved/Non_conserved* according the definition on microRNA.org
- TargetScan Folder: Target prediction results performed on TargetScan. The folder is divided in two subfolder *Conserved_Site/Non_conserved_Site* according the on TargetScan website [http://www.targetscan.org/faqs.html]

- *Sample_name*_mirdeep_result folder: Results of miRDeep2 analysis. It contains several files and folders. A detailed explanation about output files can be found on miRDeep2 website - [https://www.mdc-berlin.de/8551903/en/research/research_teams/systems_biology_of_gene_regulatory_elements/projects/miRDeep]
- Target_*Sample_Name* Folder: This folder is generated when target prediction is performed on single sample. The output files generated are similar to ones described above.
