# Supplementary material for: iMir: An integrated pipeline for high-throughput analysis of small non-coding RNA data obtained by smallRNA-Seq
Source: BMC Bioinformatics. 2013 Dec 13;14:362. doi: 10.1186/1471-2105-14-362 (PMC3878829; doi:10.1186/1471-2105-14-362)
Supplement: Additional file 3 — Additional Materials and Methods and Additional Figure Legend. [file 1471-2105-14-362-S3.pdf]

Additional Materials and Methods

Samples preparation and smallRNA-Seq. Human breast cancer MCF-7 cells were cultured either in standard culture conditions (exponentially growing cells) or following estrogen-deprivation for 5 days, a condition that leads to growth arrest of these hormone-responsive cells [42-43].
Total RNA was extracted from cell cultures with TRI-Reagent (Sigma-Aldrich, Italy) according to the manufacturer's instructions. RNA concentration in each sample was measured with a ND-1000 spectrophotometer (NanoDrop) and its integrity assessed with the Agilent 2100 Bioanalyzer by using Agilent RNA 6000 nano kit (Agilent Technologies).
Determination of miRNA expression profiles was performed by next generation sequencing (NGS) with sequencing-by-synthesis technology. 7.5µg of total RNA was used in a library preparation according to the Illumina TruSeq small RNA sample preparation protocol (Illumina, USA).
Libraries were multiplexed, by using Illumina indices set A, with indices 7 (RPI7) and 11 (RPI11) for MCF-7 cells exponentially growing and growth-inhibited respectively. Sized miRNA libraries were gel purified and sequenced on GAIIx (Illumina, USA) at a concentration of 10pM for 36 cycles, plus 7 additional cycles for indexes sequencing.
This dataset is available through GEO Dataset (Accession Number GSE40617).

Small non-coding RNAs databases. Annotated piRNA sequences were downloaded in Fasta format from NCBI Nucleotide database by searching "piRNA piR" and then by selecting INSDC (Genbank) entries for desired organism (Homo Sapiens, Mus musculus or Rattus norvegicus).
UCSC Table browser [Kuhn et al., 2009], was used to download tRNA and RefGenes sequences.
Rfam [Burge et al., 2013] was used to download non-piRNA small non-coding RNA sequences.

1. Kuhn RM, Karolchik D, Zweig AS, Wang T, Smith KE, Rosenbloom KR, Rhead B, Raney BJ, Pohl A, Pheasant M, Meyer L, Hsu F, Hinrichs AS, Harte RA, Giardine B, Fujita P, Diekhans M, Dreszer T, Clawson H, Barber GP, Haussler D, Kent WJ. **The UCSC Genome Browser Database: update 2009**. Nucleic Acids Res. 2009; **37**(Database issue):D755–761. doi: 10.1093/nar/gkn875.
2. Burge SW, Daub J, Eberhardt R, Tate J, Barquist L, Nawrocki EP, Eddy SR,Gardner PP, Bateman A. **Rfam 11.0: 10 years of RNA families**. Nucleic Acids Res 2013 Jan;41(Database issue):D226-32. doi: 10.1093/nar/gks1005. Epub 2012 Nov 3. PubMed PMID: 23125362

Additional Figure

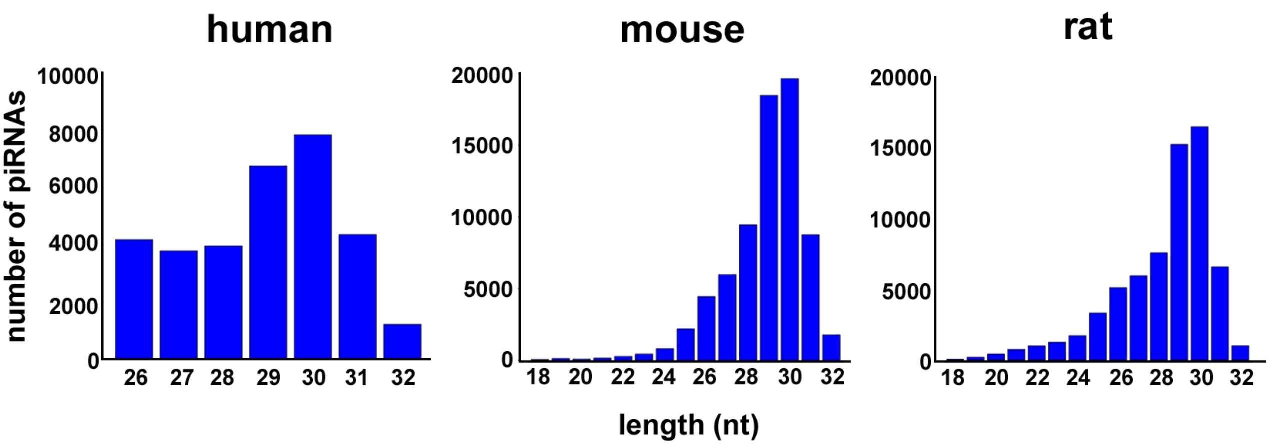

Legend: Length-distribution of known human, mouse and rat piRNAs from NCBI Nucleotide database. Histograms showing length distribution of piRNA sequences (downloaded from NCBI) for the three species included in iMir pipeline (Human, Mus Musculus, Rattus Norvegicus). X-axis shows the length of piRNAs and Y-axis the relative number of piRNAs. These histograms represent a clear peak around 26-32 bps, a characteristic of this class of sncRNA.
